# The role of the cytoskeleton in cell body enlargement, increased nuclear eccentricity and chromatolysis in axotomized spinal motor neurons

**DOI:** 10.1186/1471-2202-6-19

**Published:** 2005-03-17

**Authors:** David L McIlwain, Victoria B Hoke

**Affiliations:** 1 Department of Cell and Molecular Physiology, University of North Carolina School of Medicine, Chapel Hill, NC 27599, USA; 2 Biomedical/Biotechnology Research Institute, North Carolina Central University, Durham, NC 27707, USA

## Abstract

**Background:**

When spinal motor axons are injured, the nucleolus, nucleus and cell body of the injured cell transiently increase in size, the nucleus becomes more eccentrically placed, and the organization of polyribosomes into Nissl bodies is temporarily disrupted. The mechanisms for these classical morphological responses to axotomy have not been satisfactorily explained.

**Results:**

In this study we address the role of the cell body cytoskeleton in these structural changes. We show that the cytoskeleton of uninjured lumbar motor neuron cell bodies maintains nucleolar, nuclear and cell body size and nuclear position. When isolated, the relatively insoluble cell body cytoskeleton contains Nissl bodies and lipofuscin granules. After axotomy, protein labeling increases markedly and the cytoskeleton enlarges, increasing nucleolar, nuclear and cell body size, as well as nuclear eccentricity. Nearly all of the protein mass that accumulates in the cell body after axotomy appears to be added to the cytoskeleton.

**Conclusion:**

We conclude that axotomy causes the conjugate enlargement of the nucleolus, nucleus and cell body and increases nuclear eccentricity in spinal motor neurons by adding protein to the cytoskeleton. The change in nuclear position, we propose, occurs when cytoskeletal elements of the axon cannot enter the shortened axon and "dam up" between the nucleus and axon hillock. As a consequence, we suggest that Nissl body-free axonal cytoskeleton accumulates between the nucleus and axon, displaces Nissl body-containing cytoskeleton, and produces central chromatolysis in that region of the cell.

## Background

Injury to the axon of spinal motor neurons produces major structural changes in the affected cell body. These changes include a transient enlargement of the nucleolus, nucleus and cell body, an increase in nuclear eccentricity and central chromatolysis, which is a centrifugal disappearance of polyribosome-containing Nissl bodies. While known and explored for over a century, these morphological responses to axotomy have not been adequately explained.

It has been proposed that enlargement of the motor neuron cell body after axotomy is caused by the entry of water into the cell bodies, possibly the result of an early increase in osmotically-active hydrolytic products of macromolecules within the motor neurons [1]. On the other hand, the gradual increase in total protein and RNA observed in axotomized motor neurons cell bodies [2,3] could also play a role in their enlargement after injury. That the nucleolus, nucleus and cell body enlarge together after axotomy suggests that their sizes may be coordinated, especially since a similar scaling is observed in normal, non-injured motor neurons of increasing size [4] and in motor neurons exposed to excess growth hormone [5]. It is noteworthy that the nucleus, nucleolus and cell body do not each enlarge after axotomy in all types of neurons capable of axon regeneration, implying that an increase in size may not be required for regrowth of the axon [6].

The repositioning of the nucleus in injured motor neurons was quantified by Barr and Hamilton [1], who, like others [7,8], found that the direction of increased nuclear eccentricity in motor neurons was usually away from the axon hillock. Suggested mechanisms for increased nuclear eccentricity include a selective influx of water into the area of the axon hillock [1] and interference with axonal transport, leading to the accumulation of axonal constituents in the injured cell body [6].

Central chromatolysis also develops progressively in the region between the nucleus and axon hillock after axotomy [6]. The gradual disappearance of Nissl bodies from the perinuclear region towards the periphery of the cell body and the concomitant loss of basophilic staining of RNA occur even as the radiolabeling and total content of RNA increase [6]. Neither the molecular basis of chromatolysis nor the reason for its appearance and centrifugal spreading between the nucleus and axon hillock is known.

In this study, we first show that the size and shape of normal, non-injured frog motor neuron cell bodies are maintained by a cytoskeleton that can be isolated. We then provide evidence that axotomy increases the synthesis and total content of proteins in the cytoskeleton, which appears to alter its structure and produce nucleolar, nuclear and cell body enlargement. We further show that the cytoskeleton maintains nuclear position in the uninjured cell body and is altered by axotomy to increase nuclear eccentricity. Finally, we propose that both nuclear eccentricity and central chromatolysis result from an accumulation of axonal cytoskeleton that is impeded from entering the truncated axon.

## Results

### The cytoskeleton maintains nucleolar, nuclear and cell body size in normal motor neurons

**Figure 1:**
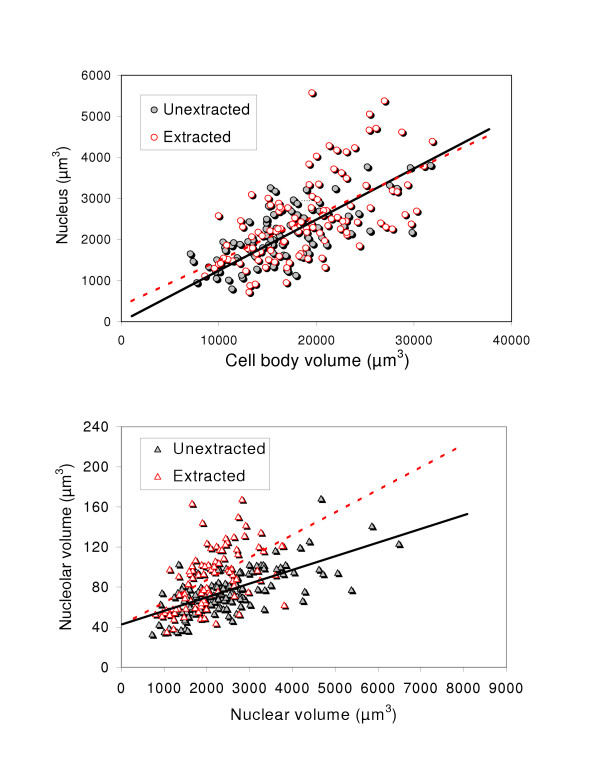
**Preservation of cell body, nuclear and nucleolar volume relationships in extracted spinal motor neurons**. Nuclear volume in individual cell bodies isolated from unfixed lumbar spinal cords of normal adult frogs scales with cell body volume (top) and nucleolar volume (bottom) before and after extraction. Significant (p < 0.01) correlation coefficients were found in first-order regression analyses for both unextracted (solid line) and extracted cells (dashed line).

**Figure 2:**
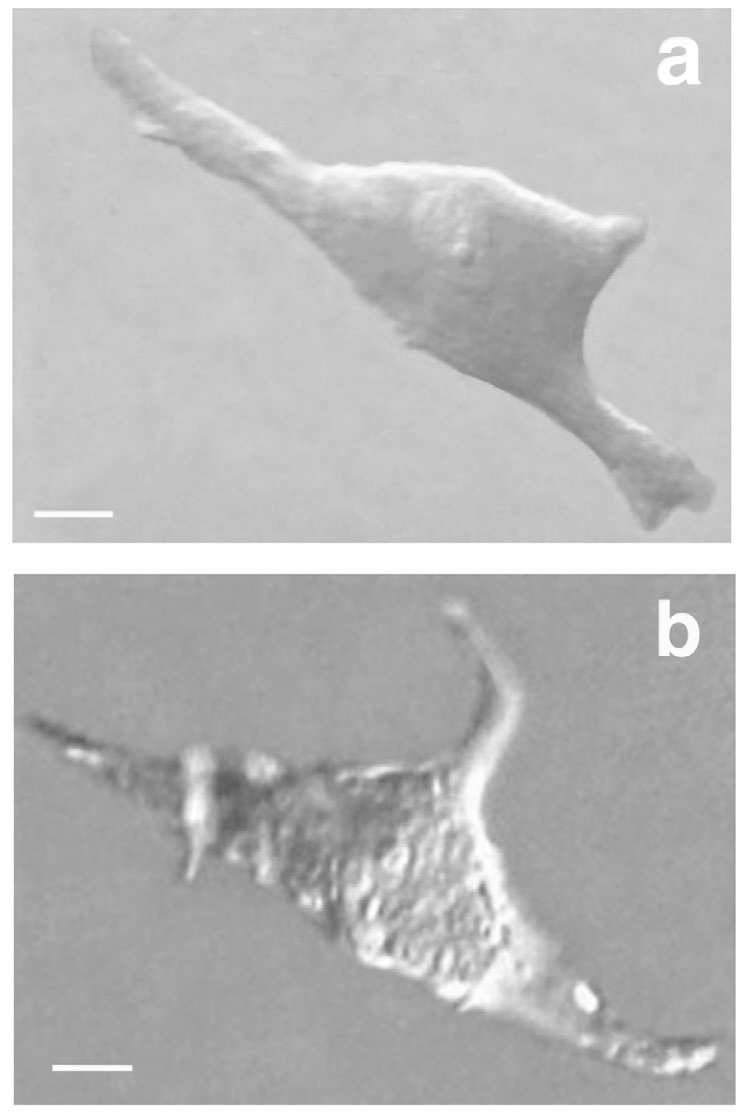
**Light microscopic appearance of extracted and unextracted spinal motor neurons**. Differential interference contrast images of lumbar motor neuron cell bodies isolated from normal adult frogs. Panel a shows an unextracted cell body. Panel b shows a different cell body isolated from normal adult frogs and extracted by a modification of the procedure of He *et al*. [12]. Each bar = 20 μm.

Isolated, unfixed motor neurons, like fixed, sectioned motor neurons, vary widely in cell body area [5,9]. The range of cell body areas in motor neurons isolated from normal animals by the method of Sinicropi and McIlwain [10] is slightly larger than that of their fixed, sectioned counterparts [5]. As with area measurements on normal motor neurons [5], nucleolar and nuclear volume increase as cell body volume increases (Fig. 1). Since the plasma membrane is damaged during the isolation of motor neuron cell bodies and is no longer semipermeable to most osmolytes [11], osmotic forces do not appear to be chiefly responsible for maintaining cell body size in isolated motor neurons. Even when intracellular membranes are permeabilized with 1% Triton X-100 and over one-half of the cell body protein is removed from isolated motor neurons by successive extraction and high salt solutions, the mean size of the nucleolus, nucleus and cell body is little affected (Table 1). This extraction procedure, which has been used to isolate the nuclear matrix and cytoplasmic skeleton of non-neuronal cells [12], reduces the cell body protein content in normal frog motor neurons from 4.35 ± 0.87 to 1.88 ± 0.29 ng/cell (a 56.8% decrease). In contrast, cell body and nuclear volume decline only by 15 and 17%, respectively, and nucleolar volume does not change significantly (Table 1). The volumes of the nucleolus and nucleus in the extracted motor neurons continue to scale with cell body volume (Fig. 1) and the extracted cells have a relatively normal appearance by differential interference contrast microscopy (Fig. 2). These findings suggest that nucleolar, nuclear and cell body size in motor neurons are maintained by cytoskeletal structures and led us to examine the ultrastructure of extracted motor neurons.

**Figure 3:**
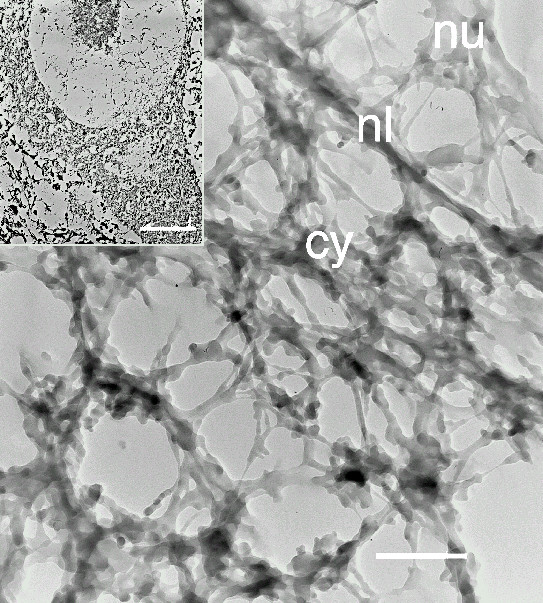
**Electron microscopic appearance of the spinal motor neuron cytoskeleton**. Embedment-free electron micrographs of two motor neurons within frog lumbar spinal cord extracted as described in Methods. The extracted spinal cord, from which the motor neurons were not isolated, was fixed with 2.5% glutaraldehyde and embedded and sectioned in DGD. After removal of the embedment medium and repeated drying through the critical point, the sections were placed on Formvar- coated grids without staining and visualized by transmission electron microscopy. Nu = nuclear skeleton; nl = nuclear lamina; cy = cytoplasmic skeleton. Inset bar = 10 μm; at higher magnification, bar = 250 nm.

Resinless thin sections [12] were prepared from frog lumbar spinal cord that had been extracted *in situ *by the same procedure used for isolated cell bodies. Isolated, extracted cell bodies were not used, because they produce friable thin sections with this embedment procedure. Transmission electron microscopy of motor neurons in unstained thin sections from which the embedment medium – diethyleneglycol distearate (DGD) – had been removed reveals a three-dimensional network of filamentous structures that remains after extraction of soluble proteins (Fig. 3). The nuclear skeleton of motor neurons is much less densely organized than the cytoplasmic skeleton, which is a highly cross-linked network of filamentous proteins with electron dense particles – possibly ribosomes [13] – interspersed throughout the lattice. The filamentous struts of the lattice are tapered, suggesting the possibility of artifactual deposition of some protein onto the lattice. These images, together with the morphometric analyses described above, indicate that the cytoskeleton is largely responsible for maintaining nucleolar, nuclear and cell body size in motor neurons.

**Figure 4:**
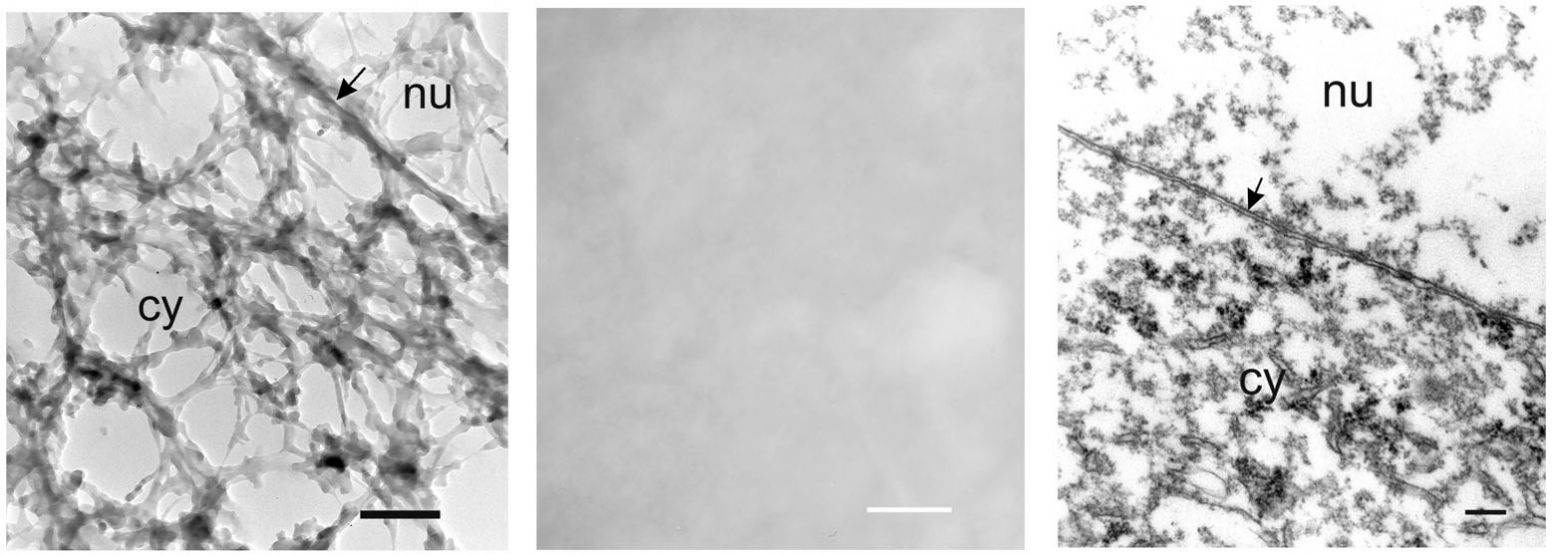
**Effect of embedment media on the ultrastructural appearance of the motor neuron cytoskeleton**. Three views of the motor neuron cell body cytoskeleton. Left: the unstained thin section from Fig. 3 of extracted spinal tissue from which DGD was removed; Middle: an unstained thin section of extracted spinal tissue from which DGD was not removed; Right: an Epon-embedded thin section of extracted spinal tissue, post-stained with uranyl acetate and lead citrate. Symbols as in Fig. 3. The arrows denote the extracted nuclear envelope. Each bar = 250 nm.

An interesting aspect of these data is the influence of the embedment medium in thin sections, which is illustrated in Fig. 4. The three-dimensional lattice seen when the embedding medium is removed is completely invisible when the embedding medium is present (Fig. 4, middle panel) and differs greatly from the two-dimensional image of stained cells embedded in plastic (Fig. 4, right panel), which masks much of the underlying filamentous structure. The obscuring influence of the embedding resin has been discussed by Penman [14].

### Nissl bodies and lipofuscin are associated with the isolated cytoskeleton

**Figure 5:**
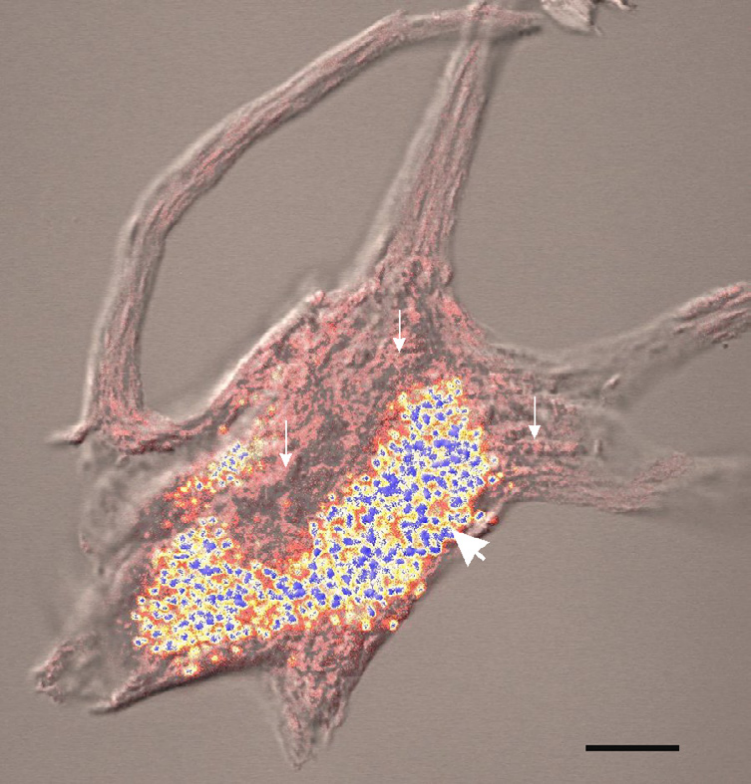
**Retention of Nissl bodies and lipfuscin by extracted motor neurons**. Confocal image of an extracted motor neuron cell body isolated from human lumbar spinal cord and stained with methylene blue. The pink structures (small arrows) are Nissl bodies and the blue and yellow structures are lipofuscin granules (large arrow). Bar = 25 μm.

Motor neurons isolated from the grass frog stain with basophilic dyes before and after extraction, indicating the presence of ribosomes. However, they do not contain well-defined Nissl bodies, such as those seen in human motor neurons. To determine whether Nissl bodies are associated with the isolated cytoskeleton, we examined cell body cytoskeletons isolated from human motor neurons and stained with methylene blue. Confocal images of methylene blue-stained cytoskeletons from normal human motor neuron cell bodies showed abundant Nissl bodies in the cell body and proximal dendrites (Fig. 5). As with isolated frog motor neurons, the appearance of isolated human cell bodies is relatively unchanged after extraction.

Adult human motor neurons, unlike those of frog, also contain large amounts of lipofuscin, and cytoskeletons isolated from human motor neuron cell bodies retain their lipofuscin (Fig. 5). The possibility that lipofuscin granules are trapped within the interstices of the cytoskeleton of the cell body and dendrites became apparent when isolated human cell bodies being examined by differential interference contrast microscopy (DIC) were exposed to SDS. When concentrations of up to 1% of the detergent were introduced at the edge of the coverslip, the cell bodies often began to swell and, one by one, individual lipofuscin granules were released and floated away from the enlarged cell body. The granules had average diameters of about 2.5 μm (range 1.8–3.0 μm, n = 16) and appeared to be insoluble in SDS. Even after depletion of most of the cell's lipofuscin, the swollen motor neuron was sometimes still visible by DIC. Lipofuscin was released only from cell bodies that underwent swelling. This sequence of events led us to infer that lipofuscin granules may be caged within the cell body cytoskeleton and can individually escape it as the lattice enlarges to pore sizes that exceed the diameter of each granule.

Although the swollen cell bodies eventually became invisible by light microscopy, the cytoskeleton may not be completely soluble in SDS. Approximately one-third of the cell body protein (31.0 ± 15.1%, n = 5) was recovered in the pellet when spun at 13,000 × *g *for 5 min in the presence of 1% SDS. This may explain our inability thus far to detect and identify any proteins in the isolated cytoskeleton by SDS-polyacrylamide gel electrophoresis.

### Axotomy enlarges the cell body cytoskeleton

**Figure 6:**
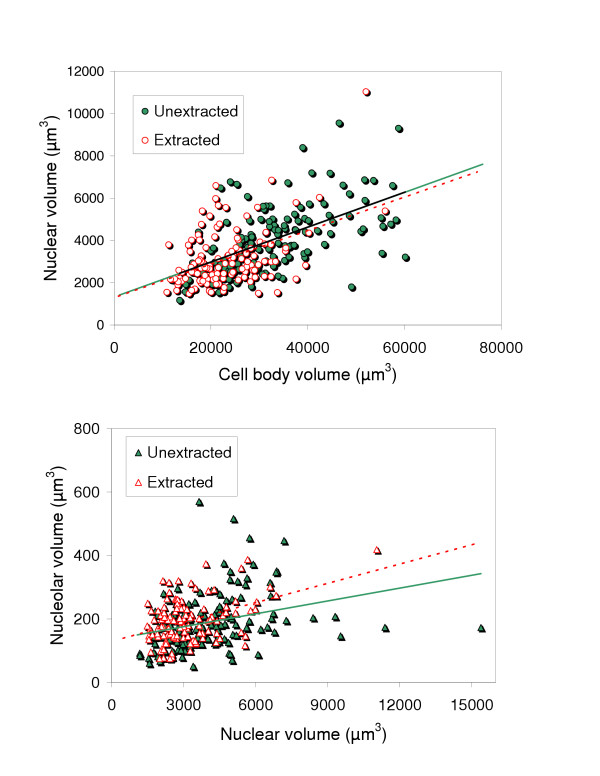
**Preservation of cell body, nuclear and nucleolar volume relationships in axotomized, extracted motor neurons**. Motor neurons enlarged by axotomy also exhibit scaling of nucleolar, nuclear and cell body volume before and after extraction. Nuclear volumes in cells isolated 16 days after transection of frog lumbar ventral roots correlate with cell body volume (top) and nucleolar volume (bottom) over a wide range of cell body size. Correlation coefficients from first-order regression analyses of unextracted (solid lines) and extracted cells (dashed lines) were significant at p < 0.05.

Transection of motor axons in the frog lumbar ventral root causes progressive enlargement of the nucleolus, nucleus and cell body of the injured motor neurons [4]. Given the evidence above that the cytoskeleton largely maintains normal nucleolar, nuclear and cell body size, one would postulate that axotomy increases their size by altering the cytoskeleton. This possibility was tested in morphometric analyses of cytoskeletons isolated from motor neuron cell bodies of frogs 16 days following ventral root transection, when injury-induced enlargement was well underway. As in normal motor neurons, the cytoskeleton of axotomized cells maintains most of nucleolar, nuclear and cell body size (Table 2). Scaling of nucleolar, nuclear and cell body sizes after injury was also evident in both unextracted and extracted cell bodies (Fig. 6). By postoperative day 16, mean nucleolar, nuclear and cell body volumes had increased 222%, 148% and 145% in the isolated cytoskeletons, respectively, *versus *254%, 159% and 164% in unextracted cell bodies (Tables 1 &2). Dividing the injury-induced increases in volume in extracted cells by those in unextracted cells, one finds that the enlargement of the cytoskeleton accounted for 91% of the increase in nucleolar size, 67% of nuclear size, and 60% of cell body size.

The injury-induced expansion of the motor neuron cell body is not uniform in all dimensions. In both unextracted and extracted cell bodies, axotomy enlarged the x, y axis much more than the z-axis of the cell. For example, in 5 experiments on a total of 145 unextracted, 16-day axotomized cell bodies, the mean radius increased 26.6 ± 4.5% after injury (p < 0.01; unpaired Student's t-test), while the mean height (z-axis) of the cells increased by only 7.3 ± 8.4% (p = n.s.). Similar differences were found in cytoskeletons isolated from 16-day axotomized cell bodies.

### Axotomy adds protein selectively to the cell body cytoskeleton

**Figure 7:**
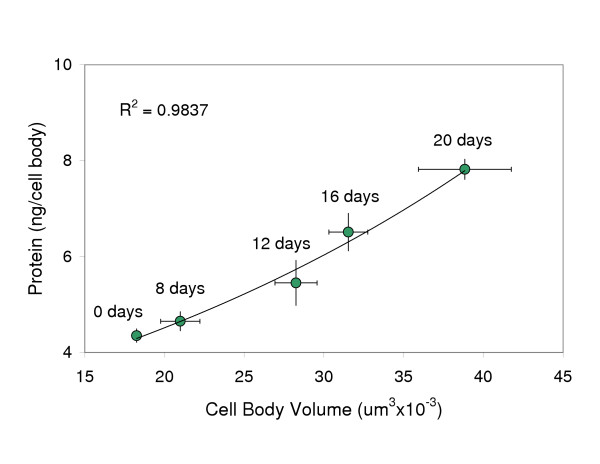
**Concurrent increases in motor neuron cell body volume and protein content after axotomy**. Injury-induced increases in mean cell body volume and protein content between 8 and 20 days after axotomy are closely correlated (p < 0.01) in third-order regression analyses. Error bars represent standard error of the mean.

Following ventral root transection, the total protein content of the injured motor neuron cell body increases steadily with increasing cell body volume (Fig. 7). When we compared the protein content of unextracted cell bodies axotomized 16 days earlier to that of their isolated cytoskeletons, we found that virtually all of the protein added to the cell body after axotomy was recovered in the isolated cytoskeleton (Table 3). These data imply that the addition of protein to the cell body cytoskeleton is responsible for its enlargement after axotomy.

When normal motor neurons were labeled *in situ *with ^3^H-leucine and their cell bodies isolated and extracted, slightly over one-half of the newly synthesized protein in normal motor neuron cell bodies was recovered in the isolated cytoskeleton (Table 4). Axotomy increased protein labeling six-fold in both unextracted cell bodies and their isolated cytoskeletons by postoperative day 16, and again about one-half of the total labeled protein was recovered in the isolated cytoskeleton (Table 4). Thus, in normal and axotomized cell bodies about one-half of the newly synthesized protein is not found in the isolated cytoskeleton. However, only new protein added to the cytoskeleton after axotomy is associated with the increase in total protein content of the cell body (Tables 3 and 4). Newly synthesized protein *not *recovered with the cytoskeleton, although elevated by injury, does not appear to contribute measurably to the increase in total protein content of the cell body after axotomy, indicating that it is exported and/or degraded more rapidly than most protein in the isolated cytoskeleton.

### Axotomy increases nuclear eccentricity by altering the cytoskeleton

Based upon evidence that the cytoskeleton maintains nuclear location in non-neuronal cells [15], we analyzed nuclear position in cytoskeletons isolated from non-injured, extracted motor neuron cell bodies. Extracted frog motor neurons showed the same degree of nuclear eccentricity found in unextracted cell bodies (Table 5). Approximately 10% of the normal cell bodies, whether extracted or unextracted, had nuclei that contacted the cell periphery (100% eccentricity). After ventral root transection, nuclear eccentricity in the isolated cytoskeleton increased to the same degree as it did in unextracted cell bodies (Table 5). Approximately 60% of the injured cell bodies had nuclei that touched the cell periphery, whether or not they were extracted. Thus, the cytoskeleton maintains nuclear position in normal motor neurons, as it does nuclear volume, and is altered by axotomy to produce an increase in nuclear eccentricity.

## Discussion

### The cell body cytoskeleton in normal spinal motor neurons

The ability to isolate individual neuronal cell bodies simplifies volumetric measurements of the entire cell body and its cytoskeleton and permits one to quantify their total protein content and radiolabeling. The isolated cell body cytoskeleton retains the shape and most of the size of the unextracted motor neuron. It maintains the size relationships among the nucleolus, nucleus and cell body over a wide range of cell body volumes and fixes the position of the nucleus within the cell.

The morphological characteristics of the isolated cytoskeleton are so typical of motor neurons *in situ*, that it is difficult to conceive that the cytoskeletons we isolate do not exist in the cells from which they are derived. However, our experimental procedures do cause changes in the living cell bodies. The somewhat smaller volumes of the nucleus and cell body in isolated cytoskeletons (Tables 1 and 2) and the tapered appearance of its filaments (Fig. 3) probably reflect those changes. Nissl bodies might not be associated with the cytoskeleton *in vivo*, although cytoskeletons isolated in a similar way from non-neuronal mammalian cells also contain polyribosomes [13,16,17], and in plant cells, labeled polyribosomes added prior to the extraction procedure were not adsorbed by the cytoskeleton [18].

### Role of the cytoskeleton in the conjugate enlargement of the nucleolus, nucleus and cell body after axotomy

The data in this study form the basis for a new explanation for the events leading to the transient enlargement of the nucleolus, nucleus and cell body after axotomy. We find that axonal injury adds protein to the cytoskeleton, likely enlarging the cell body, as well as the nucleus and nucleolus, which are integral parts of its filamentous structure. The injury-induced accumulation of proteins in the isolated cytoskeleton implies that their rate of synthesis exceeded their rate of loss from the cell body. This condition could result from an increase in their rate of synthesis, a decrease in their rate of degradation or export from the cell body or a combination of these factors. The injury-induced increase we observed in labeled protein in the isolated cytoskeleton does not distinguish among these possibilities. However, the rate of *total *protein synthesis in frog motor neurons is probably increased after axotomy, since we have previously described multiphasic increases in total transcription during this postoperative period [4]. It is unclear how much of that increase is directed towards proteins in the cytoskeletal and non-cytoskeletal fractions, but the fact that each of these two fractions receives about one-half of the total label both before and after axotomy suggests that the rate of protein synthesis increases in both fractions. We can also infer that the relationships between protein synthesis, degradation and export differ in the cytoskeletal and non-cytoskeletal fractions of the injured motor neuron cell bodies. As noted, the increase in labeled protein in the isolated cytoskeleton is accompanied by an increase in protein mass, indicating that the rate of addition of new protein to the cytoskeleton is not matched by an increase in the rate of protein loss from it. In contrast, the injury-induced increase in labeled protein that is *not *associated with the isolated cytoskeleton produces no significant protein accumulation in that fraction, indicating equal rates of protein synthesis and loss after axotomy. These observations illustrate once again why it is unsafe to use labeled protein as an indicator of protein mass.

### Are neurofilaments and peripherin major determinants of cell body size and shape in motor neurons?

An intriguing question raised by our study pertains to the identity of the cytoskeletal constituents of the filamentous lattice in spinal motor neurons. On the one hand, there is evidence that cell body size in neurons can be changed by manipulating its intermediate filament content. For example, increased cell body size in transgenic rat motor neurons overexpressing the high molecular weight component of neurofilaments (NF-H) can be reduced by concurrrently increasing the expression of the low molecular weight component (NF-L) [19]. Likewise, cell body length in differentiated PC-12 cells changes when peripherin is inhibited by transfecting the cells with small interfering mRNAs [20]. Conversely, axotomy-induced cell body enlargement is accompanied by increases in the cell body content of NF-L in frog motor neurons [10] and increased peripherin immmunoreactivity in rat spinal motor neurons [21]. Unlike neurofilament protein, peripherin mRNA also increases after axotomy [21], a finding consistent with the abovementioned possibility of an increased rate of synthesis of cytoskeletal proteins in our study. However, if neurofilaments or peripherin are constituents of the isolated cytoskeleton, we predict that they are cross-linked (Fig. 3) or otherwise modified so as to reduce their solubility.

On the other hand, there is evidence that neither neurofilament proteins nor peripherin are responsible for maintaining cell body size and shape in spinal motor neurons. In particular, Jacomy *et al. *[22] have reported that spinal motor neuron cell bodies in transgenic mice lacking the high and middle molecular weight neurofilament subunits are incapable of producing neurofilaments, but still retain their normal shape. Likewise, Larivière *et al. *[23] found that motor neurons in transgenic mice lacking peripherin also have normal-appearing cell bodies. Although compensations for losses of the individual proteins could conceivably preserve cell body structure, these observations call into question whether neurofilaments or peripherin are essential for much of the shape of normal motor neuron cell bodies. The identification of the major structural proteins in the cytoskeleton of motor neuron cell bodies will depend upon how amenable its proteins are to current modes of protein compositional analysis.

### Role of the cytoskeleton in increased nuclear eccentricity and central chromatolysis after axotomy

We also show that changes in the cell body cytoskeleton appear to be responsible for the increase in nuclear eccentricity following axonal injury. An asymmetric addition of protein to the cytoplasmic skeleton cannot alone account for the change in nuclear position, because the fraction of axotomized cells with nuclei touching the periphery of the cell body (100% eccentricity) is about six times that found in normal cells. Thus, axotomy not only enlarges the cytoskeleton, but also causes dissolution of the lattice on one side of the cell.

Additional clues to the mechanism by which the nucleus is relocated in the cell are found in the direction of nuclear displacement and the location of chromatolysis after axotomy. As mentioned, several investigators have reported that the direction of nuclear displacement is away from the axon hillock [1,7,8]. We did not test this directionality here, because the axon is routinely lost during cell body isolation. Many others have reported that the loss of Nissl bodies (*i.e., *chromatolysis) also appears first in the region of the cell between the nucleus and the axon hillock [6]. If Nissl bodies are associated with the cell body cytoskeleton *in vivo*, as they are in the isolated cytoskeleton, then one must ask why Nissl bodies are not present in the cytoskeleton added between the nucleus and axon after axotomy. There is no reason to believe that axotomy causes Nissl bodies to be removed only from the cytoskeleton added to that particular region of the cell. A more plausible explanation is that when the axon, which does not contain Nissl bodies [24], is drastically shortened by axotomy, it cannot incorporate all of the axonal cytoskeleton being synthesized by the injured cell body. Injury-induced impairment of axonal transport could exacerbate this problem. The excess of Nissl body-free axonal cytoskeleton could then accumulate between the nucleus and axon hillock, causing chromatolysis and further enlargement in that particular region of the cell body, thereby contributing to increased nuclear eccentricity away from the axon hillock. It could also act as a force for disassembly of the cytoplasmic skeleton on the opposite side of the nucleus.

Other evidence exists for "damming up" of cytoskeletal proteins destined for the axon. We have previously shown that axotomy increases the content of tubulin and neurofilament protein in the injured cell body, but decreases their content in the axon proximal to the injury site [10]. That study also showed that the volume of the axon, which is especially dependent upon neurofilaments [25,26], decreases after axotomy, while the cell body volume is increasing. Those observations, together with the data presented here, lead us to propose that axotomy causes newly synthesized axonal cytoskeleton to accumulate between the nucleus and axon hillock, resulting in an increase in nuclear eccentricity away from the axon hillock and chromatolysis between the nucleus and axon hillock.

A requirement of this proposal is that it explain the direction in which chromatolysis develops. The loss of basophilic staining after axotomy is termed "central chromatolysis" because in many instances it first appears near the nucleus and spreads outward towards the axon hillock [6]. If the axonal cytoskeleton dams up in this region of the injured cell body, why does the loss of basophilia spread centrifugally? It is not known exactly where assembly of the axonal cytoskeleton begins in normal or axotomized motor neurons. However, there is some information about the assembly of the cytoskeleton in non-neuronal cells. In her pulse-chase analysis of the incorporation of newly synthesized proteins into the cytoskeletal framework of non-neuronal cells, Fulton [27] found autoradiographic evidence that new cytoskeleton is first assembled near the nucleus, and then moves towards the periphery of the cell body. If the assembly of the axonal cytoskeleton in motor neurons follows the same pattern after axotomy, then chromatolysis could spread from the nucleus to the cell body periphery. As the movement of newly synthesized axonal cytoskeleton towards the hillock is impeded, chromatolysis would appear first near the nucleus, spreading outward towards the hillock with time and displacing and compressing the basophilic cytoplasmic skeleton. This action could produce a densely basophilic rim in the cell body periphery, as has often been observed [6]. Further accumulation of the axonal cytoskeleton could ultimately spread to other regions of the cell body.

## Conclusion

We have shown that nucleolar, nuclear and cell body volume in spinal motor neurons, as well as nuclear position, is maintained by the cytoskeleton. The isolated cytoskeleton, whose protein composition has not been determined, contains slightly less than one-half of the total cell body protein. The cytoskeleton is a filamentous lattice that is most clearly seen by transmission electron microscopy in resinless thin sections. Nissl bodies are associated with the isolated cytoskeleton, as are lipofuscin granules, which may be caged within the filamentous lattice.

Axotomy increases nucleolar, nuclear and cell body volume, largely by altering the cytoskeleton. The likely cause of the injury-induced enlargement is the addition of new protein to the cell body cytoskeleton. Axotomy also increases nuclear eccentricity by altering the cell body cytoskeleton. We postulate that both increased nuclear eccentricity and central chromatolysis in axotomized spinal motor neurons are consequences of damming up of Nissl body-free axonal cytoskeleton between the nucleus and axon hillock.

## Methods

### Surgical procedures

All ventral root transections were performed on adult southern grass frogs (*Rana pipiens*) of both sexes, purchased from Carolina Biological Supply, Burlington, NC. The left 9^th ^and 10^th ^ventral roots were transected 6–9 mm from the spinal cord *via *a dorsal laminectomy under general anesthesia (135 mg Finquel/kg body wt.). Animals were allowed to survive for up to 20 days before isolation of motor neuron cell bodies. Pre- and postoperative care and euthanasia by decapitation were conducted under institutional review in accordance with AVMA standards.

### Cell body isolation

**Figure 8:**
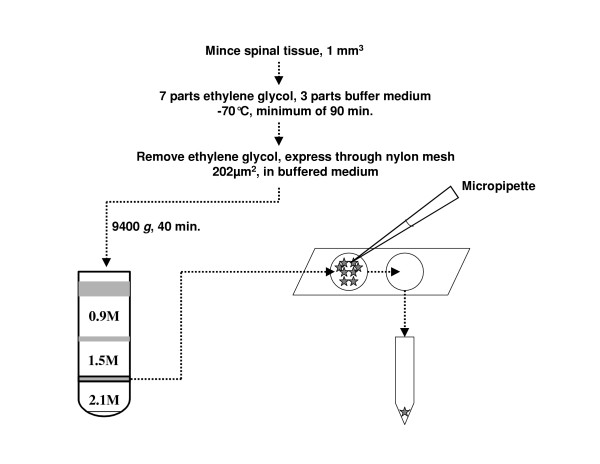
**Isolation procedure for spinal motor neuron cell bodies**. Flow chart of the procedure used to isolate individual spinal motor neuron cell bodies from the frog [10]. Nylon mesh with pore widths of 351 μm^2 ^was substituted for the isolation of human motor neurons.

Figure 8 illustrates the procedure used to isolate motor neuron cell bodies from unfixed frog lumbar spinal cord [10]. Small pieces of *R. pipiens *lumbar spinal cord were first cryoprotected with 70% ethylene glycol and frozen at -80°C. Cryoprotection improves the quality and yield of motor neurons [28] and permits storage of tissues until needed. After thawing, the tissue was suspended in 0.9 M sucrose in 1.7 mM sodium citrate buffer (pH 5.0) containing 15 mM glucose and expressed through nylon bolting cloth of 202 μm pore size. Nylon cloth with pores of 351 μm on a side was used for the larger human motor neurons. The cell bodies were then centrifuged in a discontinous sucrose gradient to obtain a crude neuronal fraction, from which individual cell bodies were removed with a glass pipette, rinsed twice in 1.5 M sucrose and pooled with other purified cell bodies. Motor neurons were identified by their distinctive size, shape and biochemical profiles [29]. In each experiment, 100–400 cells were collected from the lumbar spinal cord of three grass frogs.

### Cytoskeleton isolation

Cytoskeletons from isolated motor neuron cell bodies were obtained by serial extraction of soluble proteins, using a modification of the method of He *et al*. [12]. The buffer solution used in each step of the extraction procedure contained 50 mM HEPES (pH 7.4), 250 mM sucrose, 250 mM NaCl, 10 mM MgCl_2_, 1.2 mM phenylmethanesulfonyl fluoride, and 1 mM EGTA. The sample was exposed to 100 μl of ice-cold 0.5% Triton X-100 in extraction buffer, 35 μl of DNAase (100 μg/ml) treatment for 30 minutes at 24°C., followed by two rinses in 100 μl of chilled buffer alone, and extraction with 100 μl of chilled 0.25 M (NH_4_)_2_SO_4_, and then 2 M NaCl. After each step, the cell bodies were centrifuged at 1,000 × *g *for 3 minutes.

### Morphometry

The volume of isolated motor neuron cell bodies was quantified using images from a Leitz Diavert microscope equipped with a Fairchild CCD 3000 camera and analyzed with a Technology Resources Imagemaster 1000. Cell body volume was calculated as the product of the mean of three measurements of cell body area and the mean of the height at the center and opposite ends of the cell body. The cylindroidal shape of the cell body was determined from measurements of the z-axis in 120 cells suspended in 1.2 M sucrose and viewed between two coverslips with DIC optics. At the center of the cell body, the z-axis averaged 19.3 ± 6.5 μm and was only 102.3 ± 2.2% and 98.6 ± 4.7% of that value, respectively, at opposite ends of the cell body. In five separate experiments, the z-axis was 72 ± 7% as long as the average cell body radius. The flatness of the cell bodies was not induced by pressure from the apposed coverslips, since it was also observed in cells tumbling slowly in a droplet of buffer. Nucleolar and nuclear volumes were calculated as spheres, using the mean radius from three measurements of nucleolar or nuclear area.

Nuclear eccentricity was measured by the method of Barr and Hamilton [1], using the formula [AC÷(AB-CD)] × 100%. A-D are points along a line from the center of the cell body (A) through the center of the nucleus (C) to the cell periphery (B). AC is the distance between the centers of the cell body and nucleus (each computed from their areas by the software program). AB is the distance between the center of the cell body and its periphery and CD is the distance between the center of the nucleus and the nuclear lamina. Nuclear eccentricity of zero means the centers of the nucleus and cell body are identical, while 100% eccentricity refers to nuclei in contact with the cell body perimeter.

### Confocal microscopy

Human motor neuron cell bodies were isolated from lumbar or cervical spinal tissue obtained at autopsy from an individual without neurological disease. Cell body cytoskeletons prepared by the procedure outlined above were stained for 3 min. with 0.0032% methylene blue and examined with a Leica TCS-NT confocal microscope.

### Electron microscopy

Intact, resinless thin sections of cytoskeletal preparations from isolated motor neuron cell bodies are technically difficult to obtain. Thus, lumbar spinal tissue from adult frogs was examined after extraction by the method used for isolated cell bodies with slight modifications. Segments of longitudinally hemisected lumbar spinal tissue 1–2 mm long were exposed sequentially to HEPES-buffered 0.5% Triton X-100, 0.25 M (NH_4_)_2_SO_4 _and 2 M NaCl and fixed in 2.5% glutaraldehyde. To minimize myelin swelling, the segments were kept on ice and DNAase treatment was omitted.

Embedment was carried out at 72°C in increasing concentrations of diethyleneglycol distearate (DGD; Electron Microscopy Sciences, Fort Washington, PA, USA) from which 90% of the acetone-soluble contaminants had been removed. Thin sections were then cut on the DGD-embedded tissue, placed on Formvar-coated, carbon-stabilized grids and the DGD removed with dry *n*-butanol for 1 hour × 3 at 40°C. The resinless sections were then transferred stepwise into absolute ethanol and dried through the CO_2 _critical point after 6 wash cycles in a Balzers apparatus.

Transmission electron microscopy was performed at 80 kV with a Zeiss EM 10 CA microscope. Negatives were digitized and image contrast and brightness were adjusted electronically. In other experiments, plastic sections were cut from extracted spinal tissue embedded in Spurr's low viscosity Epon following fixation in 2.5% glutaraldehyde and 1% osmium tetroxide and were post-stained with 1% uranyl acetate and Reynolds' lead citrate.

### Protein analyses

After TCA precipitation, using a modification of the method of Bensadoun and Weinstein [30], total cell body protein content was measured by the micro method of Lowry *et al*. [31], standardized to bovine serum albumin. A total of 200 to 300 cell bodies was used for each analysis. Triplicate samples of 1.5 M sucrose from the second rinse step were taken from regions near the purified cell bodies and used as "blanks" for the protein assay.

Radiolabeling of motor neuron proteins with ^3^H-leucine prior to cell body isolation was carried out on unoperated control and 16-day axotomized frogs, using the method of McIlwain and Hoke [32]. Briefly, longitudinal sections of lumbar spinal tissue from six unoperated frogs or from the ipsilateral side of six axotomized frogs were incubated in a Yellow Springs Instrument oxygen monitoring apparatus (Yellow Springs, OH, USA) at 17°C for 16 h in frog Ringer solution containing 1 mCi/ml of ^3^H-L-leucine. After rinsing and cryoprotecting the radiolabeled tissue, 40–200 cell bodies were isolated as described above and, in some experiments, their cytoskeletons were obtained before their radioactivity was measured by liquid scintillation counting.

## Authors' contributions

DLM designed and supervised the study, participated in parts of the microscopy, and prepared the manuscript. VBH, an experienced research technician, performed most of the experiments.

## Figures and Tables

**Table 1:** The cytoskeleton maintains most of cell body, nuclear and nucleolar volume in normal motor neurons.

	Unextracted Volume (μm^3^)	Extracted Volume (μm^3^)	E/U^a^
Nucleolus	74.5 ± 11.0	86.8 ± 18.3	1.17
Nucleus	2,402 ± 423	1,968 ± 339**	0.82
Cell Body	19,387 ± 2,160	16,409 ± 1,662**	0.85

Mean volume ± S.D from 6 experiments; approximately 50 lumbar cell bodies from 3 frogs were analyzed in each experiment. Isolated cell bodies were extracted by the method of He *et al*. [12] to obtain cytoskeletons.

^a ^E/U = extracted/unextracted

**p < 0.01, Student's paired t-test, extracted *vs*. unextracted counterpart

**Table 2:** The cytoskeleton maintains most of cell body, nuclear and nucleolar volume in axotomized motor neurons.

	Unextracted Volume (μm^3^)	Extracted Volume (μm^3^)	E/U^a^
Nucleolus	189.3 ± 37.9	191.7 ± 38.8	1.01
Nucleus	3,825 ± 864	2,916 ± 513*	0.76
Cell Body	31,717 ± 4,299	23,831 ± 3,381*	0.75

Mean volume ± S.D from 6 experiments on 16-day axotomized motor neurons.

Approximately 50 lumbar cell bodies from 3 frogs were analyzed in each experiment.

^a ^E/U = extracted/unextracted

*p < 0.05, Student's paired t-test, extracted *vs*. unextracted counterpart

**Table 3:** Selective addition of protein to the cell body cytoskeleton after axotomy.

	Total Protein (ng/cell)		Added Protein (ng/cell)
	Normal	Axotomized	
Unextracted	4.35 ± 0.87	6.51 ± 2.44*	2.16
Extracted Cells	1.88 ± 0.29††	4.07 ± 0.52**†	2.19

The total cell body protein content of isolated cell bodies and their cytoskeletons is expressed as a mean ± S.D. (n = 4–9 experiments) for groups of cell bodies isolated from normal and 16-day axotomized frogs.

* p < 0.05; ** p < 0.01: axotomized *vs. *normal, Student's unpaired t-test

† p < 0.05; †† p < 0.01: extracted *vs. *unextracted, Students unpaired t-test

**Table 4:** Newly synthesized protein in isolated cell body cytoskeletons before and after axotomy.

	^3^H-leucine-labeled protein (cpm/cell body)	
	Normal	Axotomized
Unextracted cells	36.0 ± 15.4	210.6 ± 25.7**
Extracted Cells	19.8 ± 11.1†	114.8 ± 29.1**††
E/U × 100^a^	55.0%	54.5%

Mean cpm ± S.D. for cell bodies and their cytoskeletons isolated from labeled spinal cords of normal and 16-day axotomized frogs in 3 experiments.

^a ^E/U × 100 = percent new protein allocated to the cell body cytoskeleton

** p < 0.01: axotomized *vs. *normal, Student's unpaired t-test

† p < 0.05; †† p < 0.01: extracted *vs. *unextracted, Students paired t-test

**Table 5:** Nuclear position is maintained by the cytoskeleton in normal and axotomized motor neurons.

	Nuclear Eccentricity (%)^a^		
	Unextracted	Extracted	E/U^b^
Normal	54 ± 6	61 ± 4	1.13
Axotomized	84 ± 8**	86 ± 9**	1.02

^a ^Nuclear eccentricity, expressed as a mean percent ± S.D, was determined by the method of Barr and Hamilton [1]. For each mean value shown, 98–139 cell bodies were analyzed (n = 5 experiments). At 0% eccentricity, the nuclear and cell body centers are superimposed; at 100%, the nuclear perimeter contacts the cell body perimeter.

^b ^E/U = extracted/unextracted

**p < 0.01, Student's unpaired t-test, axotomized *vs. *normal
